# Supplementary material for: Candida albicans Genetic Background Influences Mean and Heterogeneity of Drug Responses and Genome Stability during Evolution in Fluconazole
Source: mSphere. 2020 Jun 24;5(3):e00480-20. doi: 10.1128/mSphere.00480-20 (PMC7316494; doi:10.1128/mSphere.00480-20)
Supplement: TABLE S1 [file mSphere.00480-20-st001.pdf]

Table S1

	Growth ability at 24 h					Growth ability at 72 h				
Line	test	t-value	df	p	evol-anc	test	t-value	df	p	evol-anc
A1	t-test	-6.85	22	<0.0001	0.09	t-test	-12.49	22	<0.0001	0.07
A2	wilcox	89	-	0.35	-0.02	wilcox	0	-	<0.0001	0.45
A3	wilcox	0	-	<0.0001	0.42	t-test	-12.96	22	<0.0001	0.64
A4	welch	-9.02	11.47	<0.0001	0.7	wilcox	0	-	<0.0001	0.84
A5	welch	-5.58	13.31	0.0001	0.21	t-test	-8.49	22	<0.0001	0.05
A6	t-test	-4.29	22	0.0003	0.27	wilcox	0	-	<0.0001	0.34
A7	t-test	-4.17	22	0.0004	0.31	t-test	-7.95	22	<0.0001	0.26
A8	welch	-6.67	11.79	<0.0001	0.67	welch	-19.22	15.01	<0.0001	0.57
A9	wilcox	96	-	0.18	0.03	welch	-11.17	12.63	<0.0001	0.69
A10	welch	1.03	13.14	0.32	-0.08	wilcox	7	-	<0.0001	0.14
A11	welch	-3.58	11.5	0.0041	0.29	welch	-8.09	12.45	<0.0001	0.74
A12	t-test	7.26	22	<0.0001	-0.18	wilcox	36	-	0.0387	0.01
A13	welch	-7.45	11.16	<0.0001	0.74	welch	-10.56	15.23	<0.0001	0.4
A14	wilcox	94	-	0.22	0.06	wilcox	21	-	0.0023	0.36
A15	welch	-7.35	12.41	<0.0001	0.72	t-test	-9.91	22	<0.0001	0.27
A16	welch	-8	14.74	<0.0001	0.58	welch	-21.39	12.27	<0.0001	0.36
A17	welch	-4.77	11.07	0.0006	0.64	welch	-18.68	14.55	<0.0001	0.99
A18	t-test	-4.92	22	0.0001	0.05	t-test	-6.34	22	<0.0001	0.03
A19	welch	-3.9	11.83	0.0022	0.42	t-test	-12.61	22	<0.0001	0.41
A20	t-test	-9.59	22	<0.0001	0.41	t-test	-11.5	22	<0.0001	0.08
