# Supplementary figures and images for: Candida albicans Genetic Background Influences Mean and Heterogeneity of Drug Responses and Genome Stability during Evolution in Fluconazole
Source: mSphere. 2020 Jun 24;5(3):e00480-20. doi: 10.1128/mSphere.00480-20 (PMC7316494; doi:10.1128/mSphere.00480-20)

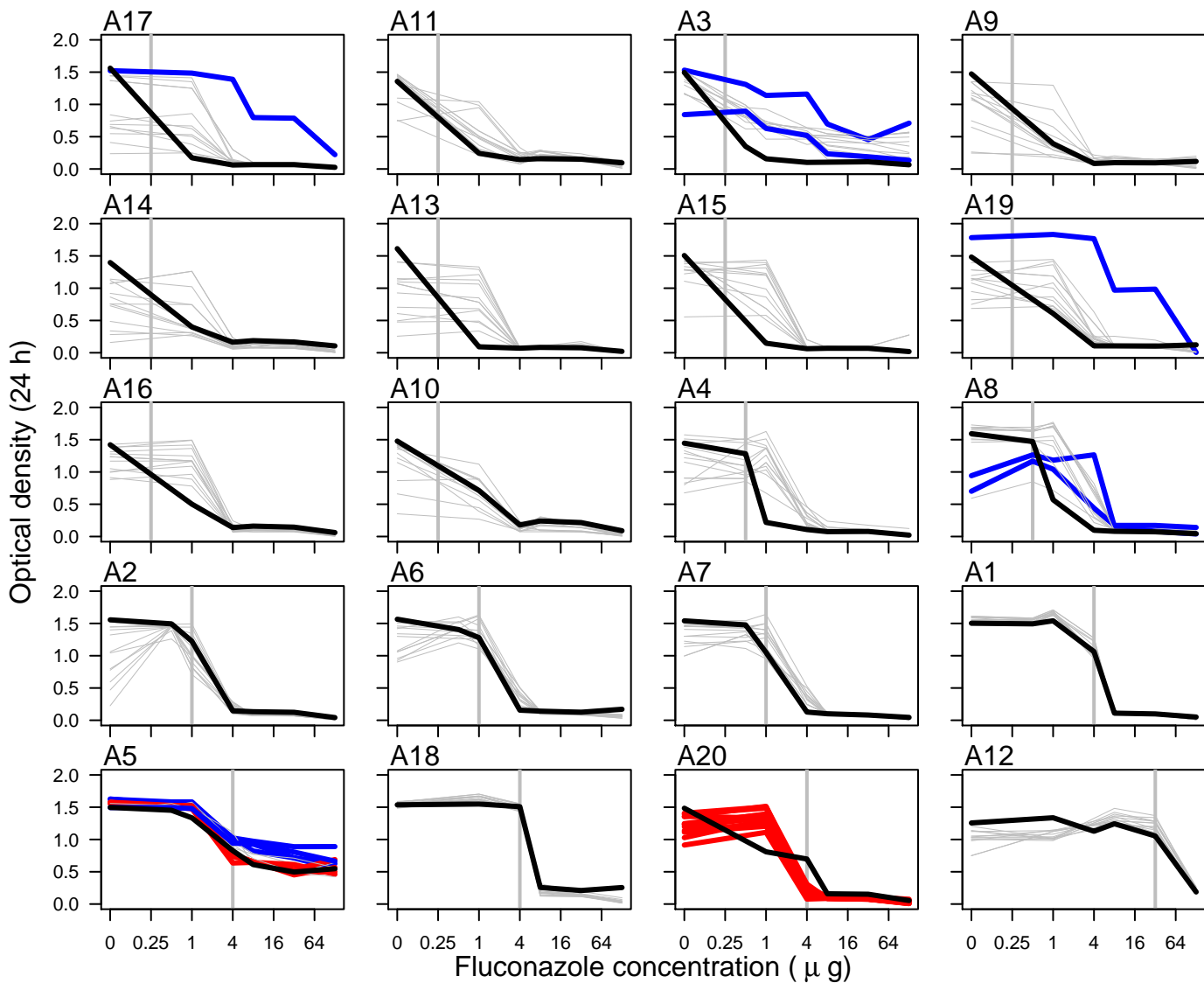

Supplement: FIG S1 [file mSphere.00480-20-sf001.pdf]

**a 24 h**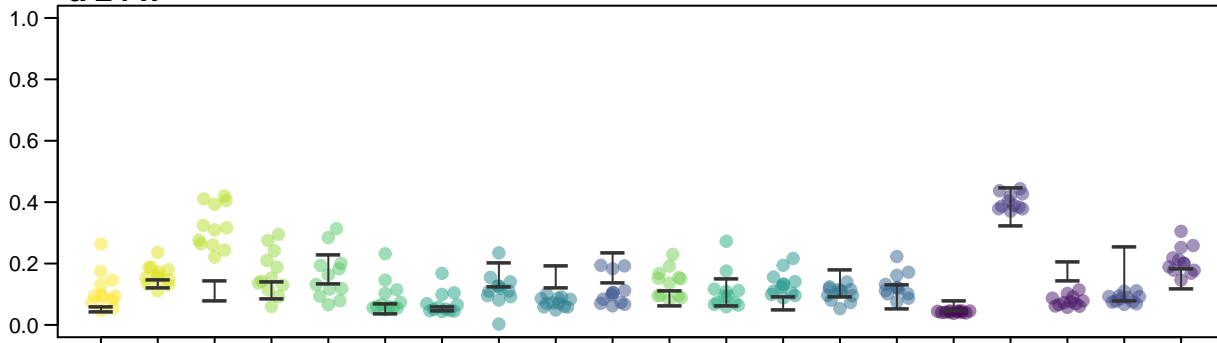**b 48 h**

Evolved tolerance

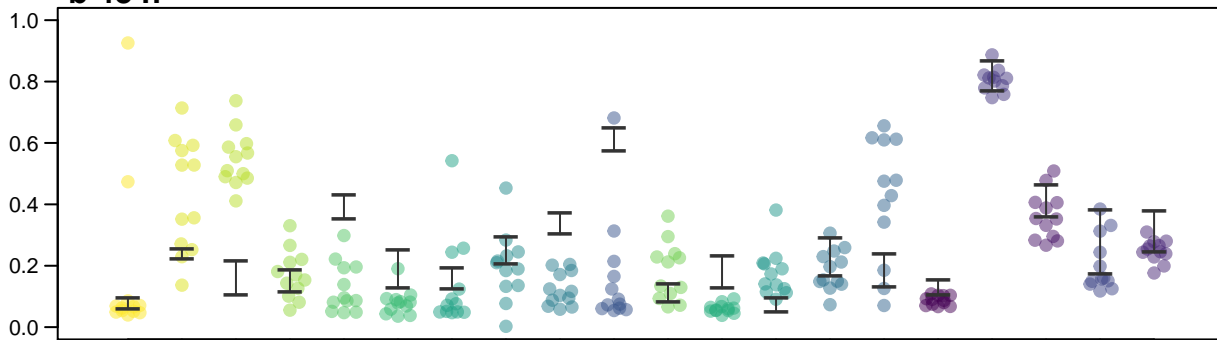**c 72 h**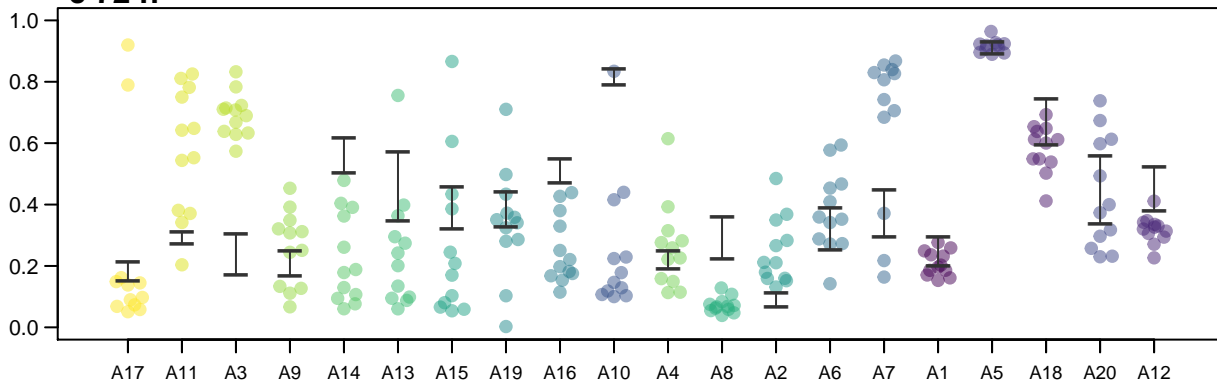

Supplement: FIG S2 [file mSphere.00480-20-sf002.pdf]

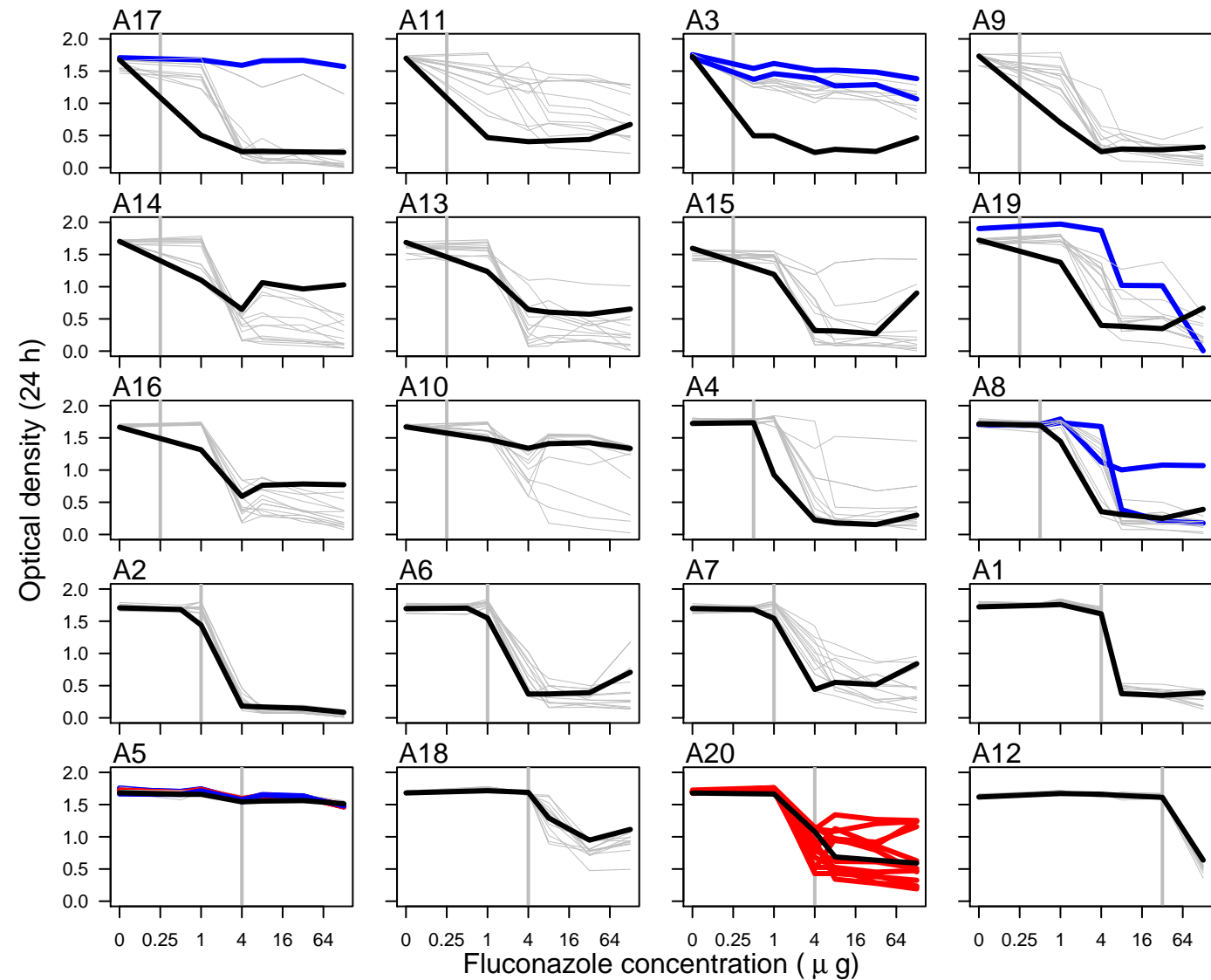

Supplement: FIG S3 [file mSphere.00480-20-sf003.pdf]

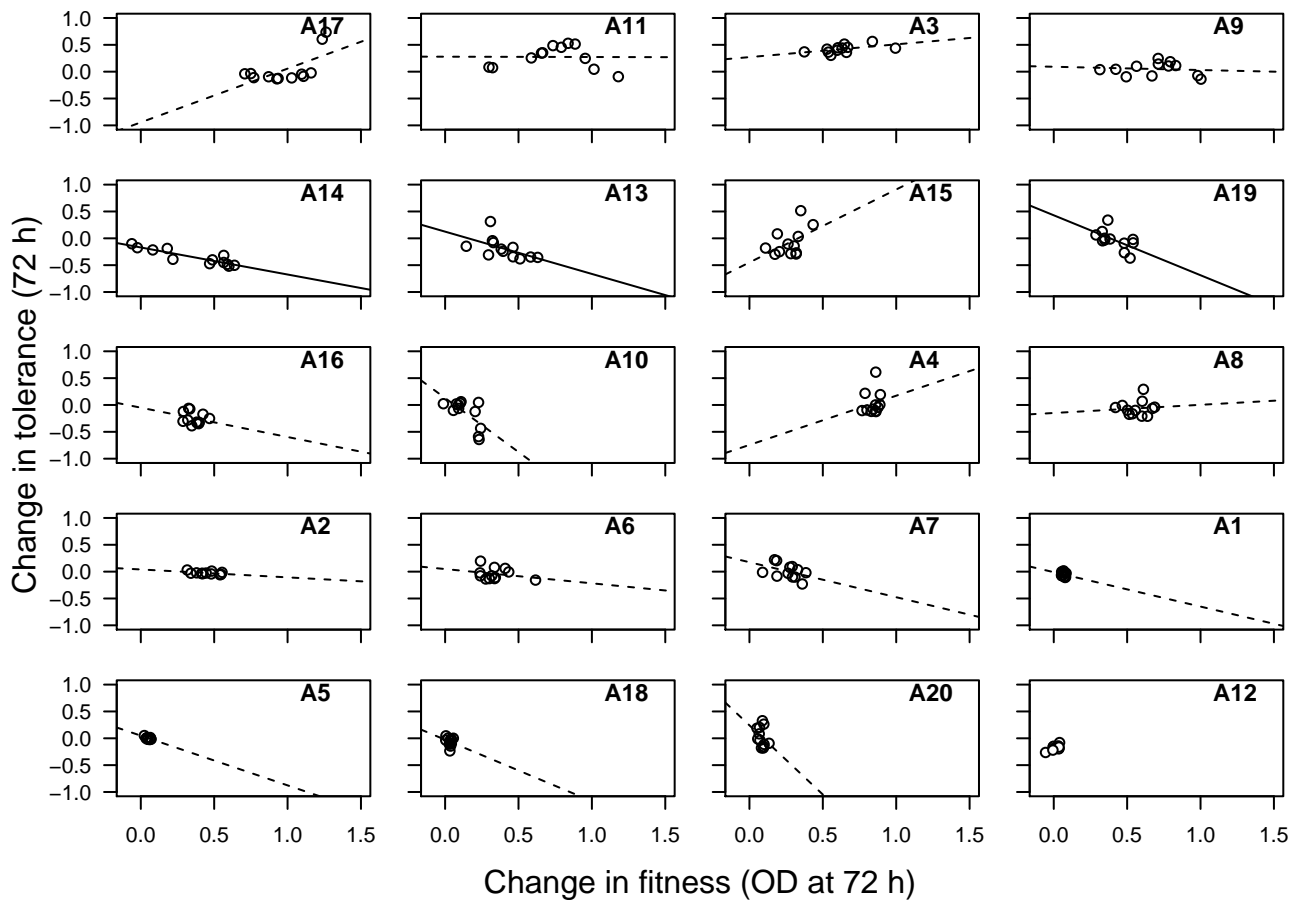

Supplement: FIG S4 [file mSphere.00480-20-sf004.pdf]

Genome size (FITC intensity)

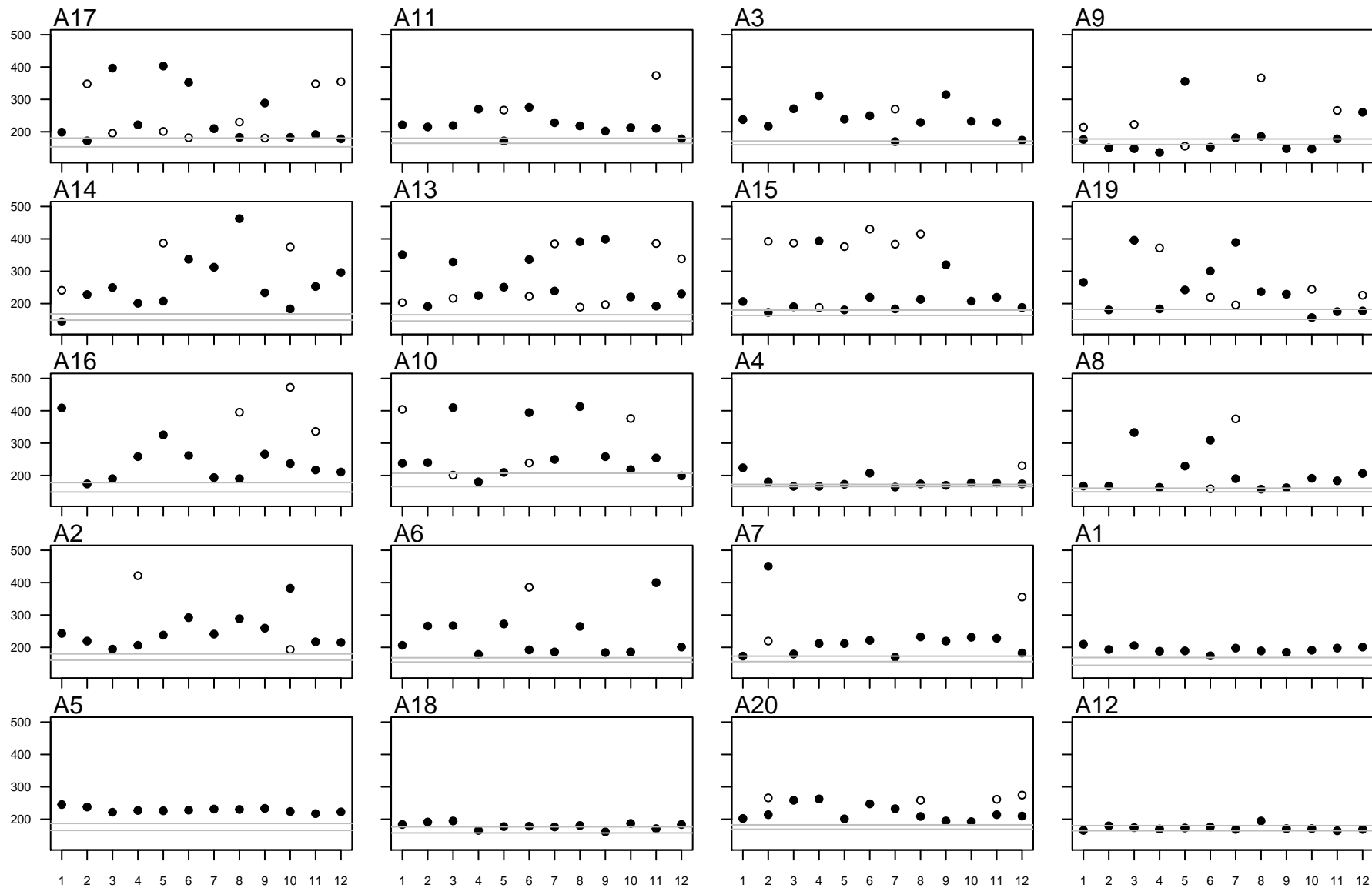

Replicate

Supplement: FIG S5 [file mSphere.00480-20-sf005.pdf]

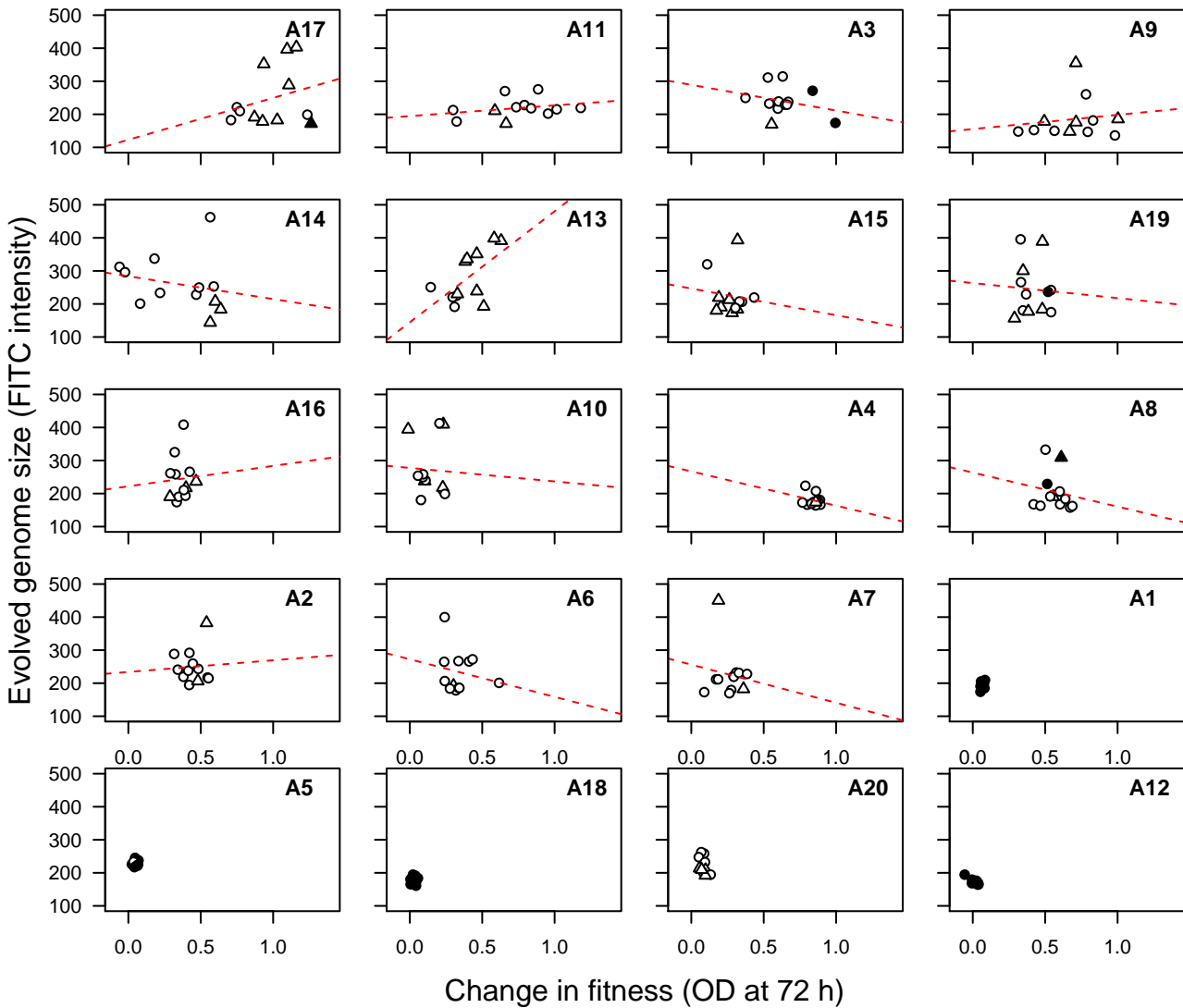

Supplement: FIG S6 [file mSphere.00480-20-sf006.pdf]

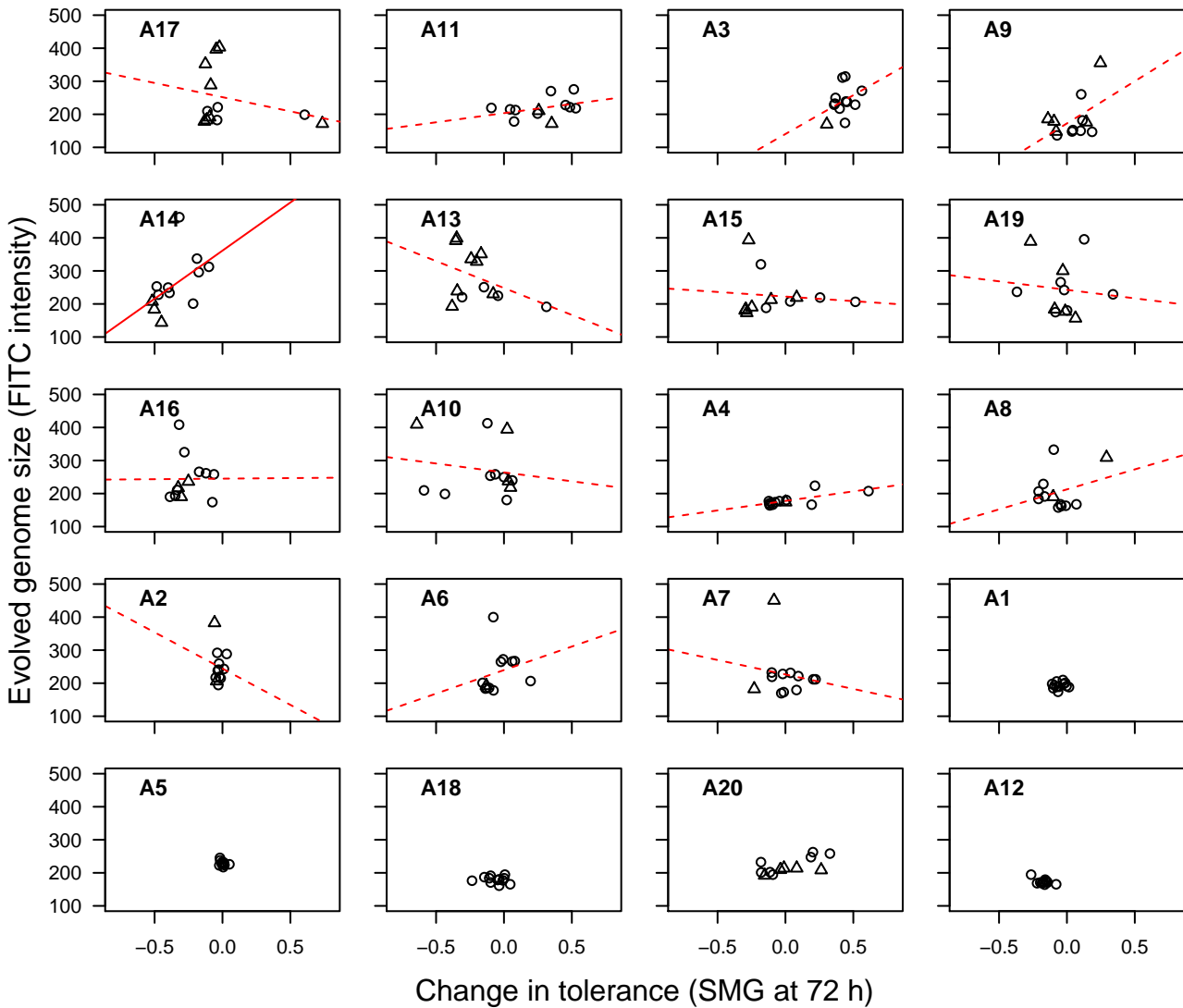

Supplement: FIG S7 [file mSphere.00480-20-sf007.pdf]
