# Supplementary material for: Candida albicans Genetic Background Influences Mean and Heterogeneity of Drug Responses and Genome Stability during Evolution in Fluconazole
Source: mSphere. 2020 Jun 24;5(3):e00480-20. doi: 10.1128/mSphere.00480-20 (PMC7316494; doi:10.1128/mSphere.00480-20)
Supplement: TABLE S2 [file mSphere.00480-20-st002.pdf]

Table S2. Minimum inhibitory concentration experiments

Exper	Time	Fluconazole level (µg)							
		0	0.5	1	4	8	32	128	512
1	Anc&Evol	x		x		x	x		
2	Anc	x		x					
3	Anc&Evol	x	x	x	x				
4	Anc	x		x	x	x	x		
5	Anc&Evol	x				x	x	x	
6	Anc&Evol	x					x	x	x
